# Assessment of Renal Function by the Stable Oxygen and Hydrogen Isotopes in Human Blood Plasma

**DOI:** 10.1371/journal.pone.0032137

**Published:** 2012-02-13

**Authors:** Tai-Chih Kuo, Chung-Ho Wang, Hsiu-Chen Lin, Yuan-Hau Lin, Matthew Lin, Chun-Mao Lin, Hsien-Shou Kuo

**Affiliations:** 1 College of Medicine, Taipei Medical University, Taipei, Taiwan; 2 Institute of Earth Sciences, Academia Sinica, Nankang, Taipei, Taiwan; 3 Department of Laboratory Medicine, Taipei Medical University Hospital, Taipei, Taiwan; 4 FU-CHEN Hospital, Taipei, Taiwan; 5 Garfield Medical Center, Monterey Park, California, United States of America; University of Tokushima, Japan

## Abstract

Water (H_2_O) is the most abundant and important molecule of life. Natural water contains small amount of heavy isotopes. Previously, few animal model studies have shown that the isotopic composition of body water could play important roles in physiology and pathophysiology. Here we study the stable isotopic ratios of hydrogen (δ^2^H) and oxygen (δ^18^O) in human blood plasma. The stable isotopic ratio is defined and determined by δ_sample_ = [(R_sample_/R_STD_)−1] * 1000, where R is the molar ratio of rare to abundant, for example, ^18^O/^16^O. We observe that the δ^2^H and the δ^18^O in human blood plasma are associated with the human renal functions. The water isotope ratios of the δ^2^H and δ^18^O in human blood plasma of the control subjects are comparable to those of the diabetes subjects (with healthy kidney), but are statistically higher than those of the end stage renal disease subjects (*p*<0.001 for both ANOVA and Student's t-test). In addition, our data indicate the existence of the biological homeostasis of water isotopes in all subjects, except the end stage renal disease subjects under the haemodialysis treatment. Furthermore, the unexpected water contents (δ^2^H and δ^18^O) in blood plasma of body water may shed light on a novel assessment of renal functions.

## Introduction

Water (H_2_O) molecule is the base of life, yet the most important molecule, making up about seventy percent of body mass. Despite the notice of importance of water, an often overlooked facet is the uniqueness of water isotopes. Natural water contains trace amount of heavy isotope hydrogen and oxygen atoms, and among which the ^2^H and ^18^O are the major ones. The ratio of ^1^H to ^2^H is about 6240 to 1, or about 155 ppm [1], [2], [3] in the V-SMOW (Vienna Standard Mean Ocean Water) water standard, and the ratio of ^16^O to ^18^O is about 499 to 1, or about 2005 ppm [4]. In terms of the molar unit, the concentrations of ^2^H and ^18^O are in the range of millimolar, which is comparable to the concentrations of many biochemical metabolites. This naturally raises an intriguing question, “Is there any biological/biomedical role played by these isotopic waters?”. However, due to a high-level complexity of human physiology, the significance of the presence of isotopic water *in vivo* remains unclear.

In general, water molecules that contain the ^2^H and ^18^O atoms (isotopic waters) are considered as behave identically as the abundant water (^1^H_2_^16^O). Accordingly, the isotopic water has been used as a marker to estimate body water content [5], [6] or the tracer of energy metabolism [7]. However, in terms of the physical and chemical properties, the isotopic waters possess subtle yet unique differences to the abundant waters [8]. This causes the isotopic variations in meteoric waters, varied by geographic locations [9], [10]. Moreover, with the unique properties of isotopic waters, the stable isotopic ratios of hydrogen (δ^2^H) and oxygen (δ^18^O) in various biological materials have been used as “atomic fossils or tracers” in paleodietary, meteorology, anthropology, ecology, and modern food-chained network [9], [11], [12], [13], [14], [15], [16].

Furthermore, several studies do indicate that the isotopic water may have profound biochemical and physiological effects. For example, the ^2^H_2_O can promote the formation of microtubules by stimulating the polymerization of tubulin subunits [17], [18], and result in cell death [19]. Vasdev and coworkers showed the increase of ^2^H_2_O content can prevent hypertension in the spontaneous hypertension rat [20]. On the other hand, the depletion of deuterium (^2^H) in the water of culture medium reduces the growth rates of different animal cell lines [21]. O'Grady and coworkers showed that the signatures of hydrogen (δ^2^H) and oxygen (δ^18^O) isotope ratios in the body water of an untreated streptozotocin-induced diabetes mellitus are distinct from those of the normal mice [22]. Thus, it is clear that the concentration of the rare isotopes (^2^H and ^18^O) in water do have biological meanings, although the cause-effect relationships and mechanism remain unknown.

Here we study and explore the possible role played by the isotopic waters in biology. We investigate relationship between the human health conditions and the stable isotope ratios of hydrogen (δ^2^H) and oxygen (δ^18^O) in human blood plasma. We observe that the δ^2^H and δ^18^O values in human blood plasma are associated with the human renal conditions. The δ^18^O in the blood plasma of the control subjects and diabetes subjects (without renal dysfunction) are 87% and 160% higher than the end stage renal disease subjects (renal dysfunction cases), respectively. The δ^2^H in blood plasma of the healthy kidney groups (the control subjects and the diabetes subjects) are 72% and 92% higher than the renal dysfunction group. However the blood plasma water contents (δ^2^H and δ^18^O) in the control subjects and the diabetes patients have no difference.

## Results

**Figure 1:**
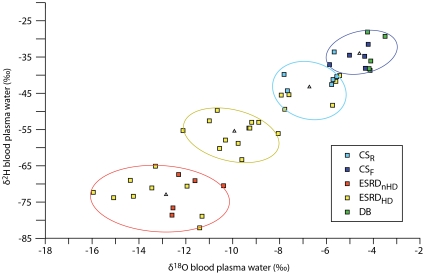
The stable isotopic ratios of hydrogen (δ^2^H) and oxygen (δ^18^O) in human blood plasma. Squares represent the distributions of the δ^2^H and δ^18^O of human blood plasma, colored by group: CS_R_ in cyan, CS_F_ in blue, ESRD_nHD_ in red, ESRD_HD_ in yellow, and DB in green. The colored circles are the clusters obtained by applying the *k*-means analysis with a preset of 4 clusters to the all datasets. The blue circle comprises all CS_F_ (100%) and all DB (100%). The cyan circle is constituted of most CS_R_ (83%) and several ESRD_HD_ (22%). The yellow circle encloses about half of the ESRD_HD_ data (44%). The ESRD_nHD_ (100%) is clustered in the red circle along with 9 ESRD_HD_ data points (33%). The triangle denotes the centroid of each cluster.

The stable isotope ratios of hydrogen (δ^2^H) and oxygen (δ^18^O) from 48 human blood plasma's water samples were measured. The samples were randomly obtained from five groups of participants, the non-fasting control subjects (CS_R_, n = 6, Table 1), the fasting control subjects (CS_F_, n = 5, Table 1), the fasting end stage renal disease subjects without haemodialysis treatment (ESRD_nHD_, n = 5, Table 2), the fasting end stage renal disease subjects with the haemodialysis treatment (ESRD_HD_, n = 27, Table 2), and the fasting diabetes subjects (DB, n = 5, Table 3). It is worth a note that this is a pilot study conducted in a small scale to explore the isotopes level in human, which allows one to carry out a large scale study later. Furthermore, to avoid any biased and carefully interpret our data, we analyzed the *entire* datasets (n = 48) by using the k-means clustering algorithm with a total number of 10,000 repeated runs. By applying the *k*-means clustering algorithm [23] with a preset of 4 clusters to the entire datasets, the four clusters are to be found as a CS_F_ plus DB cluster, a CS_R_ cluster, a ESRD_HD_, and a ESRD_nHD_ cluster (Figure 1 and Table S1). Twenty two percent of the ESRD_HD_ data locate in the CS_R_ cluster and forty four percent are within the ESRD_nHD_ cluster, indicates that the ESRD_HD_ data are dispersed between the CS_R_ and the ESRD_nHD_ clusters. In addition, the level of blood urea nitrogen (BUN), creatinine, and estimated glomerular filtration rate (eGFR) from the ESRD_HD_ patients are lower than that of the ESRD_nHD_. However, the level of δ^2^H and δ^18^O from the ESRD_nHD_ are lower than that of the ESRD_HD_ but more clustered (Table 2 and Figure 1). It should be noted that a more negative value of δ^2^H (or δ^18^O) means a lower concentration of water ^2^H (or ^18^O) than that of the international V-SMOW water standard.

**Table 1:** Isotopic values of control subject's blood plasma.

CS^a^^, ^ b	Sex	Sampling Date	Age	BUN (mg/dL)^c^	Creat (mg/dL)^d^	eGFR (mL/min/1.73 m^2^)^e^	δ^18^O (‰)^f^	δ^2^H (‰)^f^
***Nonfasting Controls (CS_R_)*** g								
1	M	Nov-07	67				−5.72	−41.2
2	M	Jul-08	55				−5.56	−40.4
3	M	Oct-08	43				−5.78	−42.6
4	M	Apr-09	29				−5.67	−33.6
5	F	Apr-09	27				−7.67	−44.3
6	F	May-09	31				−7.81	−39.8
***Fasting Controls (CS_F_)*** h								
7	F	Dec-09	39	11.0	0.7	99.54	−5.87	−37.1
8	F	Dec-09	43	15.0	0.6	116.52	−4.22	−31.5
9	M	Dec-09	45	15.0	0.7	96.62	−5.02	−34.5
10	M	Dec-09	30	14.0	0.7	134.14	−4.37	−34.8
11	F	Dec-09	41	7.0	0.6	117.68	−4.34	−38.1
Mean (std.)^i^								
CS_total_			41 (12.1)				−5.63 (1.2)	−38.0 (4.1)
CS_R_			42 (16.2)				−6.37 (1.1)	−40.3 (3.7)
CS_F_			40 (5.8)	12.4 (3.4)	0.66 (0.05)	112.9 (15.3)	−4.76 (0.7)	−35.2 (2.6)

a. The 11 human blood plasma samples (CS-1 to CS-11) were collected from the control subjects with healthy kidney.

b. All control subjects are inside the country without traveling three months prior to sample.

c. The blood urea nitrogen (BUN) test is to evaluate the renal function by measuring the amount of nitrogen in the form of urea in blood. The normal level of urea in blood is 7–20 mg/dL.

d. The creatinine is the metabolite of creatine. The creatinine test is used as an indicator of renal function. The normal level of creatinine in blood is 0.7–1.2 mg/dL for male, and 0.5–1.9 mg/dL for female [48].

e. The estimation of Glomerular filtration rate, an index of renal function. The eGFR value is calculated based on the MDRD (Modification of Diet in Renal Disease) formula, eGFR = 186×serium creatinine^−1.154^×age^−0.203^×(1.212 if black)×(0.742 if female) [49].

f. The ratio is reported as the δ-notation (‰) relative to the international V-SMOW (Vienna Standard Mean Ocean Water) standard and normalized on the scale that the δ ^18^O and δ^2^H of SLAP (Standard Light Antarctic Precipitation) are −55.5‰ and −428‰, respectively [47].

g. CS_R_'s are human blood plasma samples collected from the control subjects (CS-1 to CS-6) at random. These subjects didn't take the BUN, Creat, and eGFR test.

h. CS_F_'s are human blood plasma samples collected from the control subjects (CS-7 to CS-11) fasting for 8 hours.

i. The numbers in the parenthesis are the standard deviations taken all numbers of each group.

**Table 2:** Isotopic values of end stage renal disease (ESRD) patient's blood plasma.

ESRD^a^	Sex	Sampling Date	Age	BUN (mg/dL)^b^	Creat (mg/dL)^c^	eGFR (mL/min/1.73 m^2^)^d^	Na^+^ (mmol/L)^e^	K^+^ (mmol/L)^f^	Cl^−^ (mmol/L)^g^	δ^18^O (‰)^h^	δ^2^H (‰)^i^
***No Haemodialysis treatment (ESRD_nHD_)*** j											
1	F	Sep-09	65	40.0	1.9	28.29				−12.59	−78.6
2	M	Sep-09		77.0	4.8	12.24				−12.55	−76.6
3	F	Sep-09	71	92.0	5.9	10.12				−12.31	−67.4
4	M	Sep-09	82	42.0	2.2	30.69				−11.61	−69.1
5	M	Sep-09	70	64.0	2.8	23.93				−10.39	−70.5
***Haemodialysis treatment (ESRD_HD_)*** k											
6	F	Jul-08								−11.31	−78.9
7	F	Jul-08								−11.41	−82.1
8	F	Mar-09	54	82.0	11.5	3.69	141	4.9	96	−15.95	−72.3
9	M	Mar-09	62	87.0	9.8	5.82	135	6.5	97	−15.08	−73.8
10	M	Mar-09	69	31.07	13.5	3.94	136	4.1	94	−14.37	−69
11	M	Mar-09	60	69.0	12.5	4.44	140	4.5	100	−14.24	−73.4
12	F	Mar-09	77	38.0	5.2	8.57	139	2.4	95	−13.45	−71.1
13	M	Mar-09	53	80.0	11.7	4.9	137	4.9	98	−13.3	−65.1
14	M	Mar-09	84	55.0	9.1	5.96	135	4.4	97	−12.12	−55.3
15	M	Mar-09	64	82.0	9.9	5.71	134	5.2	97	−11.95	−70.6
16	M	Mar-09	74	113.0	10.1	5.43	137	5.8	94	−11.00	−52.6
17	F	Mar-09	46	73.0	10.4	4.29	135	5.4	98	−10.66	−49.7
18	F	Mar-09	64	70.0	8.1	5.34	125	3.6	90	−10.56	−60.2
19	M	Mar-09	79	65.0	9.0	6.12	133	4.5	97	−10.31	−57.9
20	F	Mar-09	76	51.0	7.0	6.12	136	4.2	97	−9.77	−58.8
21	M	Mar-09	81	51.0	8.4	6.59	138	3.1	99	−9.61	−63.3
22	M	Mar-09	38	120.0	16.0	3.69	138	5.0	97	−9.31	−54.6
23	F	Mar-09	94	86.0	6.3	6.60	128	5.1	9	−9.29	−53.0
24	M	Mar-09	46	85.0	11.2	5.33	135	4.4	92	−9.27	−54.6
25	M	Mar-09								−8.89	−53.0
26	F	Mar-09	55	62.0	8.6	5.15	136	5.3	96	−8.06	−56.1
27	M	Mar-09	85	19.0	7.9	7.00	132	5.9	100	−7.93	−45.5
28	F	Mar-09	77	78.0	5.7	7.71	137	4.2	97	−7.78	−49.4
29	M	Mar-09	63	66.0	12.5	4.38	139	4.8	99	−7.60	−45.4
30	F	Mar-09	60	93.0	10.0	4.26	135	3.9	93	−5.74	−48.3
31	M	Mar-09	86	105.0	11.7	4.44	131	5.2	94	−5.60	−41.7
32	M	Mar-09	66	95.0	12.3	4.42	135	5.1	97	−5.44	−40.1
Mean (std.)^l^											
ESRD_nHD_			72 (7.1)	63.0 (22.4)	3.5 (1.7)	21.05 (9.36)				−11.89 (0.9)	−72.4) (4.9)
ESRD_HD_			67 (14)	73.2 (24.6)	9.9 (2.6)	5.41 (1.27)	135(4)	5.0(1.0)	92(18)	−10.37 (2.9)	−59.1 (11.5)

a. 32 human blood plasma samples were collected from ESRD (End stage renal disease) patients, fasting for 8 hours, and are subjected into two groups, ESRD_nHD_ (end stage renal disease yet with no haemodialysis treatment) and ESRD_HD_ (end stage renal disease with receiving haemodialysis treatment).

b., c., d. The BUN, Creat, and eGFR test, all is the indicator for renal function; see Table 1 footnotes c, d, and e.

e. The concentration of sodium in blood. The normal level of sodium in blood is 135–140 mmol/L.

f. The concentration of potassium in blood. The normal blood potassium level is 3.5–5 mmol/L.

g. The concentration of chloride in blood. The normal range for chloride in blood is 98–108 mmol/L.

h., i. The isotope ratio is reported as the δ-notation (‰), see details in Table 1 footnotes c and d.

j. ESRD_nHD_ samples (ESRD-1 to ESRD-5) were collected from the ESRD patients who have not yet been the haemodialysis treatment. The ESRD_nHD_ did not perform the monovalent ion test.

k. ESRD_HD_ samples (ESRD-6 to ESRD-32) were collected from the ESRD patients who are receiving haemodialysis over 6 months. These samples were collected right before the ESRD patients to receive the haemodialysis.

l. The numbers in the parenthesis are the standard deviations taken all numbers of each group.

### The water δ^18^O and δ^2^H values of the ESRD are distinctively lower than those of the CS

The values of water δ^18^O and δ^2^H in the blood plasma of the ESRD_nHD_ show distinct characteristics from those of the CS_F_ (Figure 1). By applying the Student's *t-test* and the ANOVA statistical analysis to the ESRD_nHD_ and the CS_F_ datasets, the water contents (δ^18^O and δ^2^H) of the blood plasma in the ESRD_nHD_ and the CS_F_ show significant difference [t_0.05; 8_ = 13.78 and F_0.05; 1,8_ = 189.83 for δ^18^O; t_0.05; 8_ = 15.08 and F_0.05; 1,8_ = 227.47 for δ^2^H]. The distributions of the δ^18^O and δ^2^H in blood plasma of the ESRD_HD_ are scattered between the CS and the ESRD_nHD_ (Figure 1). The δ^18^O and δ^2^H in the blood plasma water of the ESRD_HD_ are also significantly lower than those of the CS_F_ and the CS_R_.

### The homeostasis of ^18^O and ^2^H of blood plasma resists the fluctuation of ^18^O and ^2^H levels of water sources

The δ^2^H and δ^18^O values of the rain precipitation fluctuate seasonally. Since the majority of water ^2^H and ^18^O in the blood plasma would eventually come from the rain precipitation-the source of the drinking and dietary water, it would be interesting to examine the relationships between the δ^2^H and δ^18^O values in the rain water and the plasma water.

**Figure 2:**
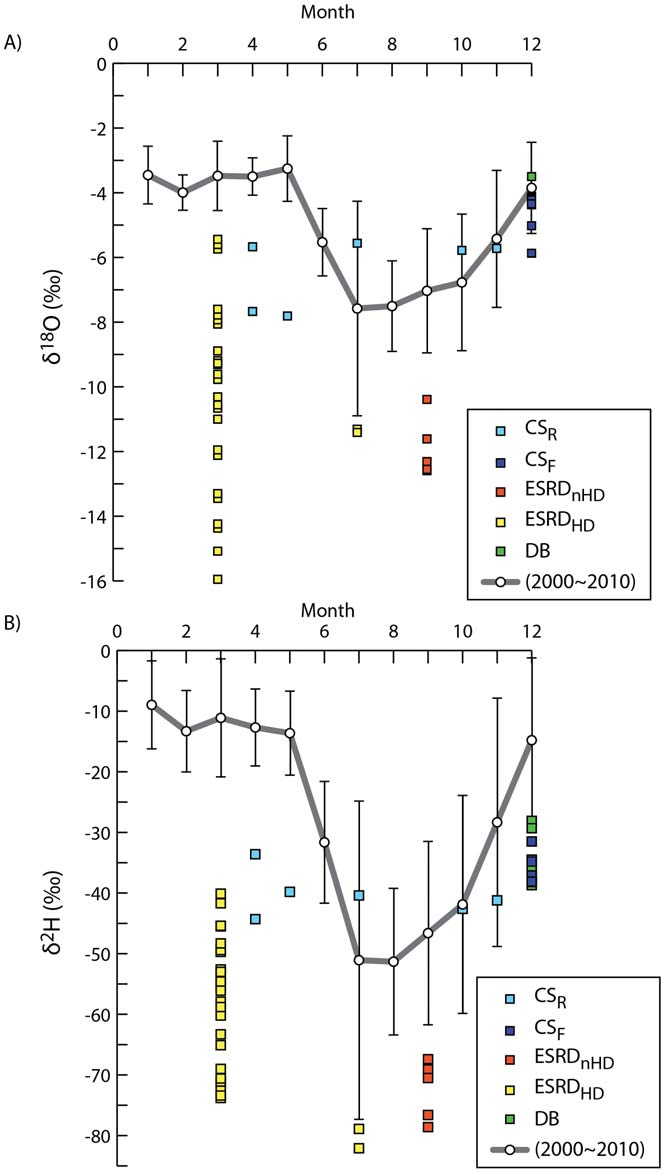
A comparison of stable isotopic water in human blood plasma and monthly mean precipitation in 2000–2010 at Taipei, Taiwan. (A) The mean values of Taipei precipitation in March, July and September from the past 11 years are idiosyncrasies to the δ^18^O in blood plasma of the ESRD_nHD_ (100%) and the ESRD_HD_ (100%). This occurrence is observed in the DB and the CS_F_. 50% of the CS_R_ are corresponding to the precipitation. (B) Both δ^2^H and δ^18^O in blood plasma of the ESRD_nHD_, ESRD_HD_, and CS_R_ are observed in a comparable manner of the monthly precipitation. None of the CS_F_ is corresponding to the precipitation. The δ^18^O and δ^2^H in blood plasma are indicated by squares, colored as in figure 1. The mean δ^18^O and δ^2^H in precipitation are indicated by circles, connected by gray lines. Error bars (black vertical lines connected by breaks) represent the standard deviation of the mean.

Here, we compared the δ^2^H and δ^18^O values of our data with those of Taipei monthly precipitation from 2000 to 2010 [24] (Figures 2A and B). From January to May, the δ^2^H and δ^18^O values of the rain water show little variation. These values start to drop in the June, reaching the minimum in the July and August and climbing back from the September to December. On the other hand, the plasma water isotope ratios in the CS group are always lower than those of the rain precipitation from January to May. However, the isotope ratios of plasma water are in the standard error of rain precipitation from July to December (Figures 2A and B). Therefore, a homeostasis of isotope δ^2^H and δ^18^O in the blood plasma of CS group is observed to against the fluctuation of daily intake water.

Due to frequent typhoons in July of Taiwan, the isotope content of rain precipitation is least but varies most among all other months. The isotope ratios of plasma water of ESRD_HD_ in July and March are significant lower than that of precipitation, again indicates a strong independency of rain water (Figures 2A and B). Although the isotope ratio of precipitation in September is close to that of CS, the same manner remains observed in ESRD_nHD_, about 35% lower than that of the precipitation. For the DB group, the δ^18^O of plasma water is comparable to that of rain precipitation in December [t_0.05; 14_ = 0.27 and F_0.05; 1,14_ = 0.07], but the δ^2^H of plasma water shows the independency of rain precipitation [t_0.05; 14_ = 3.03 and F_0.05; 1,14_ = 9.21].

### The water δ^18^O and δ^2^H values in the DB and the CS_F_ groups are similar

The values of the δ^18^O and δ^2^H from the DB are similar to those of the CS_F_. The ANOVA analysis suggests that the plasma water content (δ^18^O and δ^2^H) of the DB and the CS_F_ are comparable [F_0.05; 1,8_ = 4.63 for δ^18^O; F_0.05; 1,8_ = 0.20 for δ^2^H]. The *k*-means clustering analysis shows that the CS_F_ and the DB are similar and within the same cluster (Figure 1).

### The control subject groups (CS)

The overall mean values of the δ^2^H and δ^18^O for the CS_total_ are −5.63‰ and −38.0‰ (Table 1). The mean values of the δ^2^H and δ^18^O for the CS_R_ are −6.67‰ and −40.3‰. The means of the δ^2^H and δ^18^O for the CS_F_ are −4.76‰ and −35.2‰. From the δ^2^H and δ^18^O values of the CS_R_ and CS_F_ groups, the differences between these two groups are significant [t_0.05; 9_ = 2.88 and F_0.05; 1,9_ = 8.31 for δ^18^O; t_0.05; 9_ = 2.62 and F_0.05; 1,9_ = 6.87 for δ^2^H].

### The end stage renal disease group (ESRD)

The mean values of δ^2^H and δ^18^O for the ESRD_nHD_ are −11.89‰ and −72.44‰, and are −10.37‰ and −59.10‰ for the ESRD_HD_ (Table 2). The ESRD_HD_ (std. 2.9 for δ^18^O and 11.54 for δ^2^H) is more scattered than the ESRD_nHD_ (std. 0.9 for δ^18^O and 4.9 for δ^2^H) (Figure 1). In terms of δ^2^H, the difference between the ESRD_HD_ and ESRD_nHD_ are significant [t_0.05; 30_ = 2.5 and F_0.05; 1,30_ = 6.33]. However for the δ^18^O, the difference is insignificant [t_0.05; 30_ = 1.17 and F_0.05; 1,30_ = 1.37].

### The diabetes group (DB)

In the DB group, the mean of δ^2^H in blood plasma is −4.04‰, and is −34.08‰ for the δ^18^O. The mean fasting plasma glucose level of DB subject is 160.8±36.7 mg/dL. The creatinine level and the eGFR indicate normal kidney function of DB subjects (Table 3).

**Table 3:** Isotopic values of diabetes (DB) patient's blood plasma.

DB^a^	Sex	Age	Sampling Date	Creatinine (mg/dL)^b^	eGFR (mL/min/1.73 m^2^)^c^	glucose (mg/dL)^d^	δ^18^O (‰)^e^	δ^2^H (‰)^f^
1	F	61	Dec-09	0.6	108.38	198	−4.11	−36.1
2	F	74	Dec-09	0.5	128.54	102	−4.25	−28.1
3	F	76	Dec-09	0.6	103.58	153	−4.14	−38.7
4	F	70	Dec-09	0.8	75.81	181	−3.50	−29.3
5	M	61	Dec-09	1.0	81.01	170	−4.18	−38.2
Mean (std.)^g^		68.4 (7.1)		0.7 (0.2)	99.46 (21.46)	160.8 (36.7)	−4.04 (0.3)	−34.08 (5.0)

a. The DB blood plasma samples (DB-1 to DB-5) were collected from the diabetes patients, fasting for 8 hours.

b. The plasma creatinine concentration, an indicator of renal function.

c. The estimation of Glomerular filtration rate, an index of renal function. See Table 1 footnote e for details.

d. Fasting blood glucose level.

e., f. The isotope ratio is reported as the δ-notation (‰), see details in Table 1 footnotes c and d.

g. The numbers in the parenthesis are the standard deviations taken all numbers of DB.

## Discussion

### The water δ^18^O and δ^2^H values in the ESRD blood plasma are distinctively lower than those in the CS and the DB

In terms of the δ^18^O and δ^2^H values, the water ^18^O and ^2^H in the blood plasma of the CS and DB groups are significantly higher than those of the ESRD (*p*<0.001). The δ^18^O and δ^2^H in blood plasma of ESRD (including ESRD_nHD_ and ESRD_HD_) are 87% and 72% lower than the CS (CS_F_ and CS_R_) and are 160% and 92% lower than the DB. Thus, the values of δ^18^O and δ^2^H in blood plasma correlate with renal function in the present study.

The lowered level of water ^18^O and ^2^H in the blood plasma of the ESRD patients are intriguing, since both the normal control subjects and renal patients share the same source of drinking and dietary water. It seems that the ^2^H and ^18^O isotopes are being selectively “removed” from the water of blood plasma in patients with renal dysfunction. One of the many functions of kidney is the reabsorption of water, which is now known, at least in part, mediated by different types of renal aquaporins (AQPs), a plasma membrane protein that forms water channel [25], [26], [27]. For example, aquaporin 1 (AQP1) is localized at the proximal tubules, and descending thin limb that increases the water permeability [28]. The AQP2, AQP3, and AQP4 are localized at the collecting duct, where AQP2 is a vasopressin regulation target for the water permeability at the collecting duct [29]. Questions naturally arise whether or not these aquaporin proteins involve in the lower level of ^18^O and ^2^H in blood plasma in renal dysfunction patients. In a previous study, the molecular dynamics (MD) simulation and solution experiment of the prototypical AQP1 show that the permeability of ^2^H_2_O is similar to that of water [30], while another study shows that point mutation in the aromatic/arginine region of AQP1 allows protons pass through it [31]. Moreover, in the ESRD patients, a higher concentration of vasopressin is found [32]. Vasopressin and water deprivation accompany upregulation of AQP2 in renal collecting duct [33], [34], and downregulation of AQP2 in aging is posttranscriptional [34]. Further investigation on the level of monovalent ions such as Na^+^, K^+^, and Cl^−^ in blood plasma of ESRD_HD_ reveals that the normal functioning of active transport of ascending limb of the loop of Henle (Table 2), whereas this part of the nephron contains no aquaporins [25], [26]. Therefore, it would be interesting to find the differences of aquaporins, in terms of their biophysical, biochemical, and physiological functions in the ^2^H and ^18^O enriched water, among healthy individuals and persons with various degree of renal dysfunction. Such studies will provide clues to the observed hypo ^2^H and ^18^O in the blood plasma of people with renal dysfunctions.

### The biological isotope homeostasis of human blood plasma

It was previously shown, at the tissue level (i.e. liver, blood, nail, etc.), the nonexchangeable hydrogen isotope of quails is influenced by the deuterium-enriched dietary and drinking water [11]. Similarly, the hydrogen (δ^2^H) in human body water and hair are also affected by the intake of water and food [13]. Although it is clear that “you are what you eat”, those studies show no sign of the biological homeostasis of water isotopes. If there is no biological homeostasis of water isotopes, the isotope ratios of the water hydrogen and oxygen in the blood plasma would seasonally fluctuate with those of the rain precipitation. However, that is not what we find. In this study, the drinking and dietary water of all the participants is not artificially augmented or depleted with heavy water isotopes. Although the δ^2^H and δ^18^O values of the precipitation of Taipei city are severely associated with the asymmetric heating of island and sea during the seasonal changes [24], [35], the levels of water ^18^O and ^2^H in the blood plasma of all subjects, except the ESRD_HD_, show distinct homeostasis (Figures 2A and 2B).

**Figure 3:**
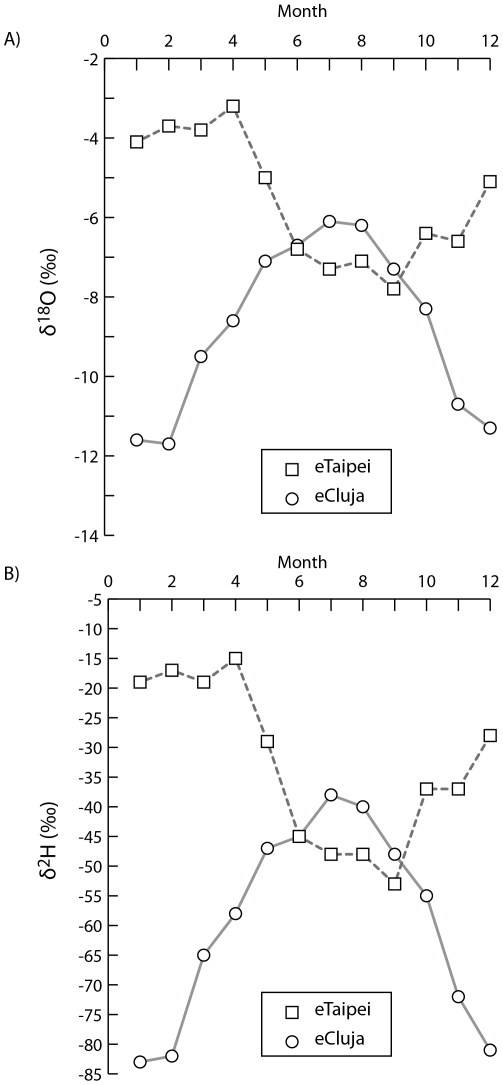
The stable isotopes of monthly precipitation in Taipei, Taiwan and in Cluj-Napoca, Romania. Shown is the estimated stable isotope of monthly precipitation, generated by using algorithm, developed by Bowen et. al. [12], [37]. The square denotes the estimated isotope of monthly precipitation in Taipei with settings of latitude 25°, longitude 121.5°, and altitude 10 m. The circle denotes the estimated isotope of monthly precipitation in Cluj-Napoca with settings of latitude 46.7°, longitude 23.6°, and altitude 335 m.

Our concept of biological homeostasis of water isotopes is coherent with the studies of Berdea et al [36]. The stable isotope δ^2^H in the blood plasma of healthy people lived in Cluj-Napoca, the fourth most populous city in Romania in Europe, is in the range of −32.3 to −38.7‰ [36], which is comparable with our CS data (Table 1). When we compare the isotope contents of monthly precipitation in Cluj-Napoca and in Taipei using the algorithm, developed by Bowen et. al. [12], [37], the isotope precipitation patterns between the two cities are distinct (Figure 3). Apparently, in the two cities, people intake very different sources of drinking and dietary water, in terms of the water isotope contents. However, the biological isotope homeostasis in human blood plasma of healthy people in Taipei in Asia is consensus of that in Cluj-Napoca in Europe. We believe that the human homeostasis is a fundamental mechanism to maintain the high level complex physiological functions.

It appears that there is a fine tune of the biological homeostasis of water isotopes reflecting differential renal conditions. For example, the ESRD_nHD_ and the normal renal groups (the CS and the DB) have different homeostasis levels. Note that the three groups with normal renal function (Tables 1 and 3), the δ^18^O and δ^2^H levels of the CS_F_ and DB are similar, yet higher than those of the CS_R_ group (Figure 1). The difference between these groups are that the CS_F_ and DB are under an 8-hr fasting while the CS_R_ group is not. In addition, we notice that the age of the CS subject (range from 27 to 67 yr.) is independence of the DB and ESRD subjects (*p*<0.001). However, the age of the DB is highly correlated to that of the ESRD (*p* = 0.93). The stable isotopic values of δ^18^O and δ^2^H in blood plasma of the ESRD_HD_ are scattered between CS and ESRD_nHD_ (Figure 1). The lack of the ^18^O and ^2^H homeostasis in the ESRD_HD_ group could be due to the times and durations of each haemodialysis, and the hydration status of different ESRD_HD_ subjects [38]. Therefore, we suggest that the levels of δ^2^H and δ^18^O in blood plasma of renal dysfunction patients may need to be monitored during the haemodialysis treatment.

### Is the hypo stable isotope of blood plasma a cause or a consequence of renal dysfunction?

In rat, when about 30% of the body water is replaced by ^2^H_2_O, histologic examination shows demolition of renal tubules but the morphology of glomerular remains unchanged [39]. Another study with the ^2^H_2_O replacement in rats has shown changes in renal function that both the glomerular filtration rate and renal plasma flow decrease. Recovery of renal function is achieved by the restoration of ^1^H_2_O [40]. Despite the above studies were carried out in rats, evidently, heavy water is toxic to the kidney as it could damage the renal tubules and change the renal function. On the other hand, since the renal patients share the same drinking and dietary water with the healthy people, we cannot attribute the renal dysfunction to the presence of the heavy water isotopes.

In fact, the causes of renal dysfunction are usually very complicated, including medications, diabetes mellitus, hypertension, sepsis, personal life style, and so on. Nonetheless, an impaired kidney causes the accumulation of metabolites, which may result in the replacement of ^2^H for ^1^H and ^18^O for ^16^O. Such replacement forms stronger chemical bonds (more stable) between the heavy atoms (from water) and light atoms (from metabolites) and thus, the isotopes level in water becomes lower. Moreover, the fluid retention caused by renal dysfunction would affect the total water flux (TWF), an important factor to monitor the isotope level of water that is highly correlated to levels of water intake and urine output [22], of human compartments. All these could eventually lead to the hypo isotope of blood plasma. In the present study, we show that the renal dysfunction is associated with the much reduced δ^18^O and δ^2^H in human blood plasma. We observe a signature of hypo isotope of blood plasma, exhibits in renal dysfunction patients but not in healthy persons or diabetes patients, although the age of diabetes subjects are correlated to that of renal dysfunction subjects. We urge further biochemical and biophysical assessments to figure out the hypo isotope of blood plasma in the ESRD patients.

As of today, the serum creatinine has become the most widely used marker in estimating the glomerular filtration rate (GFR) of kidney function. Despite the GFR estimation is based on the chronic renal disease model, this model underestimates the GFR in healthy population but overestimate in patients with impaired kidney [41], [42]. In addition, the serum creatinine used to evaluate renal function is encumbered by association with sex, age, muscle mass, race, and diet [43]. Our data and results suggest that the δ^18^O and δ^2^H of blood plasma is sensitive to the renal function but seems insensitive to age, race and diet, probably due to the biological isotope homeostasis. In sum, this pilot study along with biological data forms the base for further investigations on the hypo isotope of blood plasma in renal dysfunction patients, and opens up the possibility of using level of δ^18^O and δ^2^H in blood plasma as a potential marker for renal dysfunction. Therefore, a large scale quantitative study on the hypo isotope of renal dysfunction patients are currently planned to conduct.

### Summary

The stable isotopic ratios of hydrogen (δ^2^H) and oxygen (δ^18^O) in human blood plasma are biological isotopic homeostasis in the CS, the ESRD_nPD_, and the DB. The water status (δ^18^O and δ^2^H) of blood plasma of the CS and the DB are comparable, but is significantly distinct from the ESRD. The unexpected water in blood plasma of the human body could provide an insightful index to assess the condition of the human kidney.

## Materials and Methods

### Ethics Statement

The Taipei Medical University Institutional Review Board approved this study (approval ID: TMUH-02-09-01). The informed consent from all subjects involved in this study was not obtained as the data were analyzed anonymously. The ethics committee approved this procedure.

### Participants

According to the biochemical parameters and doctor descriptions on the participants' records, the participants were classified into three categories, the control subject (CS), the patients diagnosed with the end stage renal disease (ESRD), and the individuals with diabetes yet without detectable renal dysfunction (DB). All the DB subjects are under diabetes control. All forty eight subjects (11 CSs, 32 ESRDs, and 5 DBs) are native Taiwanese, live in the Taipei city. They had not traveled abroad for at least three months prior to the sampling date. Thus we can eliminate the isotope ratio variations caused by the geographical isotopic compositions of food and water [10], [11], [15], [16].

### Water samples

3 ml of human blood plasma sample is stored in a 15 ml falcon tube. The tube is then placed into a pre-dried vacuumed round bottle flask with 15 g of CaCl_2_ granule (Sigma-Aldrich). The round bottle flask is then capped and sealed carefully to make sure no water in air gets into the flask. The flask is incubated at room temperature for CaCl_2_ to absorb water from the human blood plasma sample for seven days. The water sample (about 2 ml) is obtained from the hydrated CaCl_2_ by vacuum distillation (Buchi Glass Oven B-585, Kugelrohr).

### Determination of hydrogen (δ^2^H) and oxygen (δ^18^O) in human blood plasma

The assessment of hydrogen (δ^2^H) and oxygen (δ^18^O) in the water samples was conducted as the following. The stable oxygen isotopic compositions were analyzed by the well-known CO_2_–H_2_O equilibration method [44], [45]. The equilibrated CO_2_ gas was measured by a VG SIRA 10 isotope ratio mass spectrometer. The hydrogen isotopic compositions were determined on a VG MM602D isotope ratio mass spectrometer after reduction of water to H_2_ using Zinc shots made by Biogeochemical Laboratory of Indiana University [46]. All isotopic ratio results are reported as the δ-notation (‰) relative to the international V-SMOW (Vienna Standard Mean Ocean Water) standard and normalized on the scale that the δ ^18^O and δ^2^H of SLAP (Standard Light Antarctic Precipitation) are −55.5‰ and −428‰, respectively [47]. The analytical precisions expressed as 1σ for the laboratory standards are better than 1.3‰ for δ^2^H and 0.08‰ for δ^18^O, respectively. The average differences of duplicate analyses of water samples are ± 1.5‰ for δ^2^H and ± 0.11‰ for δ^18^O, respectively.

### Date Analysis

To very carefully interpret our data and avoid any biased, we first analyzed the entire datasets (n = 48) by using the k-means clustering algorithm with a total number of 10,000 repeated runs. The ANOVA and the Student's t-test were performed by using the STASTISTICA 8.0 (StartSoft. Inc., Tulsa, OK). The *k*-means clustering was performed in the MATLAB R2011a (MathWorks. Inc., Natick, MA) that data are partitioned into a preset of four clusters. Euclidean distance is measured to compute the centroid of cluster, a mean of the data points within that cluster. A total number of 10,000 repeated clustering processes were performed, as a new set of initial cluster centroid position was given in each round. This procedure returns the best solution of a four clusters that each cluster is with the lowest value of sum of point-to-centroid distances.

## Supporting Information

Table S1 The partition table of the whole dataset (n = 48) into the 4 preset groups (clusters) and the centroid of each cluster are obtained via the *k-means* clustering algorithm with a total number of 10,000 repeated runs.
(DOCX)
