# Supplementary material for: Assessment of Renal Function by the Stable Oxygen and Hydrogen Isotopes in Human Blood Plasma
Source: PLoS One. 2012 Feb 13;7(2):e32137. doi: 10.1371/journal.pone.0032137 (PMC3278461; doi:10.1371/journal.pone.0032137)
Supplement: Table S1 — The partition table of the whole dataset (n = 48) into the 4 preset groups (clusters) and the centroid of each cluster are obtained via the k-means clustering algorithm with a total number of 10,000 repeated runs. (DOCX) [file pone.0032137.s001.docx]

Table S1. The partition table of the whole dataset (n = 48) into the 4 preset groups (clusters) and the centroid of each cluster obtained via the *k-means* clustering algorithm with a total number of 10,000 repeated runs.

| **δ^18^O** | **δ^2^H** | **Cluster #** |  | **Cluster #** | **Centroid of each cluster** | |
| --- | --- | --- | --- | --- | --- | --- |
|  |  |  |  |  | **δ^2^H** | **δ^18^O** |
| -5.72 | -41.2 | 4 |  | 1 | -12.8936 | -72.75 |
| -5.56 | -40.4 | 4 |  | 2 | -9.90417 | -55.7583 |
| -5.78 | -42.6 | 4 |  | 3 | -4.51545 | -34.5455 |
| -5.67 | -33.6 | 3 |  | 4 | -6.60273 | -43.5182 |
| -7.67 | -44.3 | 4 |  |  |  |  |
| -7.81 | -39.8 | 4 |  |  |  |  |
| -5.87 | -37.1 | 3 |  |  |  |  |
| -4.22 | -31.5 | 3 |  |  |  |  |
| -5.02 | -34.5 | 3 |  |  |  |  |
| -4.37 | -34.8 | 3 |  |  |  |  |
| -4.34 | -38.1 | 3 |  |  |  |  |
| -12.59 | -78.6 | 1 |  |  |  |  |
| -12.55 | -76.6 | 1 |  |  |  |  |
| -12.31 | -67.4 | 1 |  |  |  |  |
| -11.61 | -69.1 | 1 |  |  |  |  |
| -10.39 | -70.5 | 1 |  |  |  |  |
| -4.11 | -36.1 | 3 |  |  |  |  |
| -4.25 | -28.1 | 3 |  |  |  |  |
| -4.14 | -38.7 | 3 |  |  |  |  |
| -3.5 | -29.3 | 3 |  |  |  |  |
| -4.18 | -38.2 | 3 |  |  |  |  |
| -11.31 | -78.9 | 1 |  |  |  |  |
| -11.41 | -82.1 | 1 |  |  |  |  |
| -15.95 | -72.3 | 1 |  |  |  |  |
| -15.08 | -73.8 | 1 |  |  |  |  |
| -14.37 | -69 | 1 |  |  |  |  |
| -14.24 | -73.4 | 1 |  |  |  |  |
| -13.45 | -71.1 | 1 |  |  |  |  |
| -13.3 | -65.1 | 1 |  |  |  |  |
| -12.12 | -55.3 | 2 |  |  |  |  |
| -11.95 | -70.6 | 1 |  |  |  |  |
| -11 | -52.6 | 2 |  |  |  |  |
| -10.66 | -49.7 | 2 |  |  |  |  |
| -10.56 | -60.2 | 2 |  |  |  |  |
| -10.31 | -57.9 | 2 |  |  |  |  |
| -9.77 | -58.8 | 2 |  |  |  |  |
| -9.61 | -63.3 | 2 |  |  |  |  |
| -9.31 | -54.6 | 2 |  |  |  |  |
| -9.29 | -53 | 2 |  |  |  |  |
| -9.27 | -54.6 | 2 |  |  |  |  |
| -8.89 | -53 | 2 |  |  |  |  |
| -8.06 | -56.1 | 2 |  |  |  |  |
| -7.93 | -45.5 | 4 |  |  |  |  |
| -7.78 | -49.4 | 4 |  |  |  |  |
| -7.6 | -45.4 | 4 |  |  |  |  |
| -5.74 | -48.3 | 4 |  |  |  |  |
| -5.6 | -41.7 | 4 |  |  |  |  |
| -5.44 | -40.1 | 4 |  |  |  |  |
